# Motor Imagery EEG Classification Based on Transfer Learning and Multi-Scale Convolution Network

**DOI:** 10.3390/mi13060927

**Published:** 2022-06-10

**Authors:** Zhanyuan Chang, Congcong Zhang, Chuanjiang Li

**Affiliations:** College of Information, Mechanical and Electrical Engineering, Shanghai Normal University, Shanghai 200234, China; 1000479121@smail.shnu.edu.cn (C.Z.); licj@shnu.edu.cn (C.L.)

**Keywords:** brain-computer interface, motor imagery, transfer learning, data alignment

## Abstract

For the successful application of brain-computer interface (BCI) systems, accurate recognition of electroencephalography (EEG) signals is one of the core issues. To solve the differences in individual EEG signals and the problem of less EEG data in classification and recognition, an attention mechanism-based multi-scale convolution network was designed; the transfer learning data alignment algorithm was then introduced to explore the application of transfer learning for analyzing motor imagery EEG signals. The data set 2a of BCI Competition IV was used to verify the designed dual channel attention module migration alignment with convolution neural network (MS-AFM). Experimental results showed that the classification recognition rate improved with the addition of the alignment algorithm and adaptive adjustment in transfer learning; the average classification recognition rate of nine subjects was 86.03%.

## 1. Introduction

**Figure 1:**
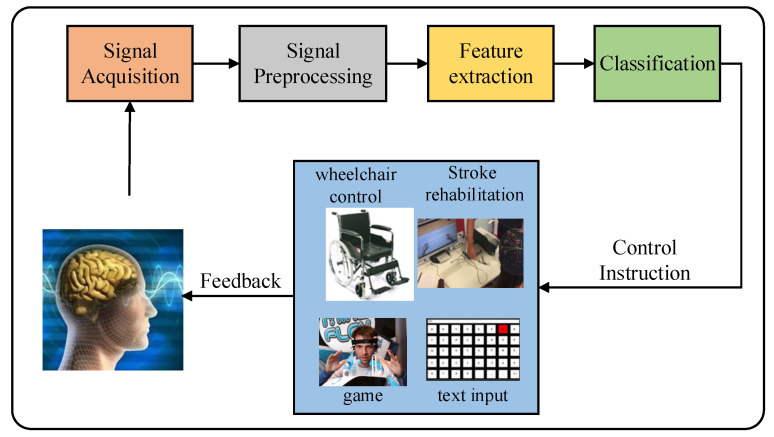
Composition of BCI System.

Brain-computer interface (BCI) systems are used to establish a connection between a brain and a computer; the computers are controlled by monitoring the activities of electroencephalography (EEG) signals. With the rapid development and promotion of BCI technology, the research on BCI technology has become the focus of attention in the field of brain science and biomedicine. BCI systems based on motor imagery are a popular research topic, and these have considerable application potential in games and nerve rehabilitation, among other fields [1,2,3,4]. BCI system usually consists of five parts: signal acquisition, signal and processing, feature extraction, classification and recognition, and control feedback (see Figure 1). The classification results are converted into control commands to control external devices. The premise of the stable application of the system is the correct decoding of EEG signals. Therefore, more in-depth research on EEG signal processing and pattern recognition will provide a more valuable reference for the wide application of BCI and play a certain role in promoting BCI technology to benefit human beings.

Deep learning, a branch of machine learning, simulates the operation of the human brain by establishing complex interconnected neural structures, and building a general model that can deal with various data. Deep learning is an end-to-end method that does not rely on manual design feature extraction methods; it can learn a large number of necessary parameters and detect valuable information [5]. Compared with support vector machine (SVM), linear discriminant analysis (LDA), and extreme learning machine (ELM), deep learning requires more model training time and hardware resources; however, computing devices such as graphics processing unit (GPU) can solve these problems to a large extent [6,7,8]. Several contemporary studies have demonstrated that deep learning is superior to contemporary classification methods, and considerable success has been achieved in deep learning-based image processing, speech recognition, and natural language processing (NLP), among other fields.

With the development of deep learning, the end-to-end deep learning method is used to process the EEG signals of motor imagery, and different network structures are designed to adapt to the classification of EEG signals of motor imagery, which meets the requirements of extracting effective features and has higher robustness. An et al. [9] applied deep belief networks (DBNs) for the recognition of imaginary left and right hand movements and obtained better network classification results than SVM. In Ref. [10], a compact convolutional neural network (EEGNet) was proposed to design a BCI system that used deep convolution and separable convolution to construct the EEG feature extraction model; EEGNet demonstrated sufficient robustness in learning multiple interpretable features in a series of BCI paradigms. In Ref. [11], a one-dimensional convolution neural network and long-short-term memory (LSTM) network were combined; EEG data were found to be highly correlated in time and less correlated in space. First, one-dimensional convolution was used to extract the time-domain features, and then, LSTM was input to further extract the time-domain features for the classification of motor imagery tasks, obtaining higher classification accuracy. In Ref. [12], mobile inception EEGNet (MI-EEGNet) was proposed; the network was divided into three modules. Inspired by EEGNet, the first module adopted separable convolution and deep convolution. Inspired by Inception [13], MobileNet [14], and Xception [15], the second module used parallel pipes for data processing; each pipe extracted features using convolution kernels of different sizes, spliced the outputs of each pipe, and aggregated data with additional convolutions. Finally, global pooling and softmax were used for classification to avoid overfitting and complexity arising from the use of full connection layers.

To overcome the differences in EEG data between subjects and the limitation of adjusting algorithms to deal with different data sets, multi-scale convolutional neural networks have been proposed for motion imagination, and these have shown good results. Dai et al. [16] proposed a mixed-scale convolutional neural network (hybrid-scale CNN, HS-CNN); the network was based on the best convolution scale that varies with the subjects. First, the best scale was selected, and the best scale convolution operation was performed on the θ, μ, and β bands (4–7 Hz, 8–13 Hz, and 13–32 Hz, respectively) to further improve classification accuracy. A multi-scale network (Auxiliary Multi-scale Input CNN for EEG, AMSI-EEGNet) was proposed by Riyad et al. [17]; it comprised four modules, namely, a pooling module, a time-space convolution module, an aggregation convolution module, and a classification module. The branch module was used to save and process each band to obtain optimal classification results. Donglin et al. [18] proposed a multi-scale fusion convolution neural network based on attention mechanism (MS-AMF) to improve the ability to learn local details of the brain region. First, the network divided the original EEG data according to different brain regions, and then, it extracted the spatio-temporal multi-scale features from these regions. The dense fusion strategy was used to maximize the information flow, and the attention mechanism was added to improve network sensitivity. Compared with the baseline method, the network has a better classification effect, so as to verify the effectiveness of the network.

The size of the data and the quality of the data limit the effect of deep learning, and EEG signals due to its complex acquisition process, and higher acquisition conditions, resulting in less practical training data. In recent years, some scholars have proposed various isomorphism and heterogeneous transfer learning methods to solve the problem of fewer EEG signals. Zhang et al. [19] proposed an instance-based transfer learning framework, which does not change the feature space and properties of motion imagery task signals, measures the similarity between the source domain and target domain signal features by perceptual hash algorithm, calculates the transfer weight coefficient, and extends the training set of the target domain. Later, Zhang and his team [20] proposed to use all the data of other subjects to train the model on the basis of Deep ConvNets network, fine-tuning and adaptive pre-training model with a small amount of target data, and compared the transfer and adaptive adjustment results of each layer of the network.

To solve the differences among individual EEG signals and the problem of less EEG data, as well as to explore a more universal deep learning method, in this study, a multi-scale convolution network is proposed based on an attention mechanism; further, a transfer learning data alignment algorithm is added to explore the application of transfer learning in the analysis of motor imagery EEG signals. The data set 2a of BCI Competition IV is used to verify the proposed network (dual channel attention module migration alignment with convolution neural network, MS-AFM). Experimental results show that adding the alignment algorithm and adaptive adjustment in transfer learning improves the classification recognition rate, and the average classification recognition rate for nine subjects reaches 86.03%.

## 2. Methods

### 2.1. Pre Processing

In machine learning, one of the main assumptions is that training data and test data belong to the same feature space and obey the same probability distribution. If this distribution changes, most statistical models need to use newly collected training data to retrain from scratch. In many real-world applications, it is expensive or challenging to obtain the required training data and reconstruct the model, that is, the need and workload of collecting training data again should be reduced. To achieve this, transfer of knowledge or learning between task domains is necessary. During EEG signal processing, due to the individual differences between subjects, these hypotheses of machine learning cannot be fully met. Typically, this problem is addressed by calibrating the session for a long time to collect high-quality and large amounts of EEG data sets; however, this depends on subjects’ cooperation and the quality of signal acquisition, greatly limiting the practical application of BCI [21,22,23]. To solve the cross-subject problem of transfer learning, in [24,25], the data in Riemannian space and European space, respectively, were aligned and transformed, which resulted in similar data distribution of different subjects, significantly improving the problem of transfer learning. Compared with Riemannian Alignment (RA), Euclidean Alignment (EA) has the advantages of fast calculation speed and unsupervised. Therefore, in this study, EA is used to calibrate all samples before data instance transfer so that the sample data have similar distribution after transformation. EA algorithm is calculated as follows:

Let us assume that there are n trials, then, the reference matrix $\bar{R}$ is calculated as follows:

(1) $$\bar{R}=\frac{1}{n}\sum_{i=1}^{n}X_{i}X_{i}^{T}$$

where $X_{i}$ is the *i*th trial, and the reference matrix $\bar{R}$ is the average of all test covariance matrices. Thereafter, the following alignment is used:

(2) $${\tilde{X}}_{i}={\bar{R}}^{-1/2}X_{i}$$

Then, the average covariance matrix of all calibrated tests is given as follows:


(3) $$\begin{array}{ll} \frac{1}{n}\sum_{i=1}^{n}{\tilde{X}}_{i}{\tilde{X}}_{i}^{T} & =\frac{1}{n}\sum_{i=1}^{n}{\bar{R}}^{-1/2}X_{i}X_{i}^{T}{\bar{R}}^{-1/2}\\ & ={\bar{R}}^{-1/2}(\frac{1}{n}\sum_{i=1}^{n}X_{i}X_{i}^{T}){\bar{R}}^{-1/2}\\ & ={\bar{R}}^{-1/2}\bar{R}{\bar{R}}^{-1/2}=I \end{array}$$

The average covariance matrix of all subjects before and after data alignment is equal, which shows that the distribution of the covariance matrix from different subjects is more similar. The aligned data can be directly used for transfer learning.

### 2.2. Signal Classification

**Figure 2:**
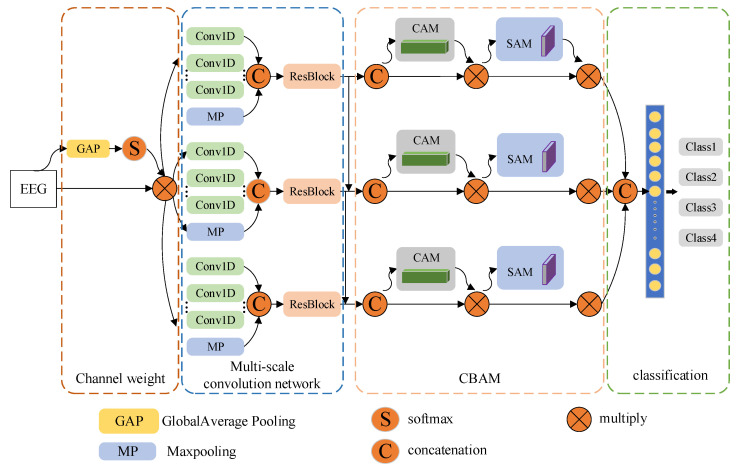
Overall architecture of the attention mechanism-based multi-scale convolution network. (channel attention module, CAM; spatial attention module, SAM).

To classify motor imagery EEG signals, we propose a multi-scale convolution network based on an attention mechanism. The proposed multi-scale convolutional neural network based on attention mechanism is a parallel three-way cascade network; it comprises four modules, namely, a channel weight calculation module, a multi-scale convolution module, a convolutional block attention module (CBAM) attention mechanism module, and a classification module. The network structure is referred to as multi-scale attention convolutional neural network (MACNN, Figure 2). Typically, channel selection is carried out manually, or it is assumed that different channels have the same effect on recognition performance. In the MACNN structure, channel weight is calculated via the global average pooling and softmax operation to avoid personal subjectivity because of the manual selection of channels and the problem that the main channel is not sufficiently prominent. The obtained channel weight is multiplied by the channel data corresponding to the original EEG, and each channel data contributes according to its own importance; as a result, the subsequent analysis is more accurate and efficient. The multi-scale convolution module mainly comprises multi-scale one-dimensional convolution and residual parts. With increasing network depth and the influence of different scale convolutions, some details are easily lost. Therefore, cascade operation is used after the residual module to fuse the convolution results of different scales as the input of the subsequent attention module. After the calculation results of each scale branch are spliced, the full connection layer is input for classification, and the final classification results are obtained from the classification module.

#### 2.2.1. Multi-Scale Convolution Network

In the field of image processing, during the actual image processing, for the same image, convolution kernels with different sizes have different receptive fields as well as performance. Therefore, by using convolution kernels with different sizes for images with different scales, better results can be achieved. Similarly, during the processing of motor imagery EEG signals, differences in the physiological structures of subjects, as well as the non-linearity and non-stationarity of potential spontaneous activity signals in motor imagery, there are considerable differences among EEG signals collected by different subjects and those collected by the same subject at different time periods. Therefore, a single convolution scale network has a better classification effect on the data of specific subjects, while the data classification results of other subjects are limited. The proposed multi-branch cascade multi-scale convolution network is used to extract the characteristics of different scales to adapt to the differences between signals of different subjects or the same subject.

The multi-scale convolution network module includes a combination of inception and residuals of three parallel branches; the inception module is inspired by the Inception module in the classical GoogLeNet. The inception part of a single branch of the network has four parallel branches, three convolution branches, and one pooling branch. After each convolution, batch normalization (BN) layer and activation layer (rectified linear unit function, ReLU) operations are carried out; the size of the three convolution cores is 1×10, 1×20, and 1×30, respectively. The inception convolution kernel size of the other two branches in the multi-scale convolution network module is 1×60, 1×70, and 1×80 and 1×150, 1×170, and 1×200, respectively. The convolution kernels of different scales are convoluted along the time axis, and the convolution results are sent to the residual module.

To prevent degradation of the network, a residual module is added after the multi-scale convolution of each branch, which also increases the network depth. Similarly, there are BN layer and ReLU operations after each convolution. The calculation results of the residual part and convolution part are combined, that is, output characteristics of the shallow layer are transmitted to the deep layer, which prevents network degradation.

#### 2.2.2. Attention Mechanism

In deep learning, the attention mechanism is used to focus on critical points among varied feature information; it ignores unimportant feature information. A CBAM is a lightweight general module. It focuses on the relationship between channels and spatial dimensions and automatically learns the importance of channels and spatial features; also, it can be integrated into any CNN architecture. Compared with the SE module which only focuses on the relationship between channels (squeeze-and-excitation), the CBAM module shows better results. Therefore, in this study, the CBAM module is added to the proposed network structure; the features calculated by the multi-scale convolution network module are further refined by giving as input to the CBAM attention mechanism module.

**Figure 3:**
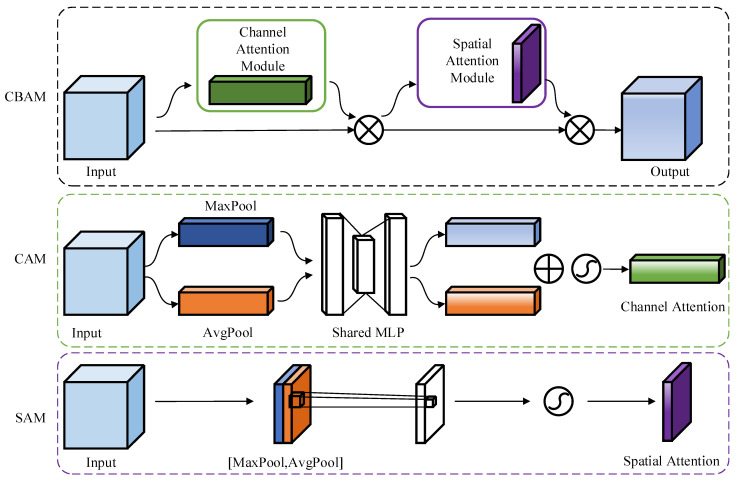
CBAM attention mechanism module.

The overall structure and submodule structure of the CBAM attention mechanism module are shown in Figure 3. It comprises two sub-modules, namely, a channel attention module (CAM) and a spatial attention module (SAM). First, the CAM performs the maximum pooling and average pooling of the input features in parallel, and then, it obtains the channel weights through the two-layer full connection layer. The results of the weighted features and the original input features are multiplied and used as input features for the SAM. First, the SAM performs the maximum pooling and average pooling along the channel dimension and splices the pooling results into the channel dimension. Then, it obtains the spatial attention features by convolution and sigmoid; then, the original input feature and output feature of the SAM submodule are multiplied and given as input for the next layer. The CBAM calculates the weights of channel and space according to the input middle layer features, and it adaptively adjusts the middle layer features by multiplying these weights. In this study, calculation results of the CBAM are directly used as the input of the classification module.

## 3. Results and Analysis

This experiment uses Python 3.9 and the deep learning keras framework (https://keras.io/, accessed on 29 September 2020) for algorithm implementation; NVIDIA Titan Xp128 G GPU is used for network model training and testing in the Linux system.

### 3.1. Introduction of Experimental Data Set

The data set used is BCI Competition IV data set 2a (http://bbci.de/competition/iv//#dataset2a, accessed on 5 May 2021), it is provided by the Department of Medical Informatics, Institute of Biomedical Engineering, Graz University of Technology (TU Graz, Graz, Austria). The dataset comprises EEG data of nine subjects. The experimental paradigm employed visual cues; subjects completed four motor imagery tasks, namely, motor imagery of the left hand, right hand, feet, and tongue. Each subject recorded data for two sessions on separate days; each session consisted of six sets of trials with 48 motor imagery sessions (12 motor imagery sessions per category), with a short rest after each session; thus, there were 288 motor imagery trials in one session and 576 motor imagery sessions in two sessions.

### 3.2. Experiments and Results

The proposed MACNN structure adopts ReLU as the activation function, which can speed up the training speed of a neural network. In MACNN training, L2 regularization and dropout technology are used to prevent the overfitting of neural network training. The L2 regularization parameter and dropout probability are set to 0.01 and 0.8 respectively, and the learning rate is set to 0.0001; Adam algorithm is used as the optimization method.

Transfer learning is used to solve the problem of less training data for a single subject. When the data are aligned, the covariance matrices of the samples can be regarded as unit matrices, and then, the data distribution can be considered similar; this will result in the best state of transfer learning theory. In this study, five modes were tested experimentally, namely, the original target experiment (original), direct model transfer (TL), model transfer with target data fine-tuning (TL + FT), data alignment processing model transfer (EA + TL), and data alignment processing model transfer with target data fine-tuning (EA + TL + FT), and the results are shown in Table 1. Compared with the direct classification results of the original network model, the average accuracy of classification results with transfer learning alignment and target data fine-tuning increases from 80.76% to 86.03%. For the proposed MACNN structure in this paper, although the number of deep learning training parameters increases and the amount of convolution calculation increases by using multiple scales and branches, it can adapt to different subjects and improve the classification accuracy of subjects’ data. However, the method of direct data transfer may lead to lower recognition accuracy. Adding a small amount of target data for self-adaptive adjustment results in a small improvement. Data alignment is a preprocessing operation before model training. The EA method makes the sample data distribution more similar, and at the same time, the calculation is simple, which can bring better results for subsequent sample transfer. After data alignment preprocessing and adding a small amount of target data for self-adaptive adjustment, the results are significantly improved.

**Figure 4:**
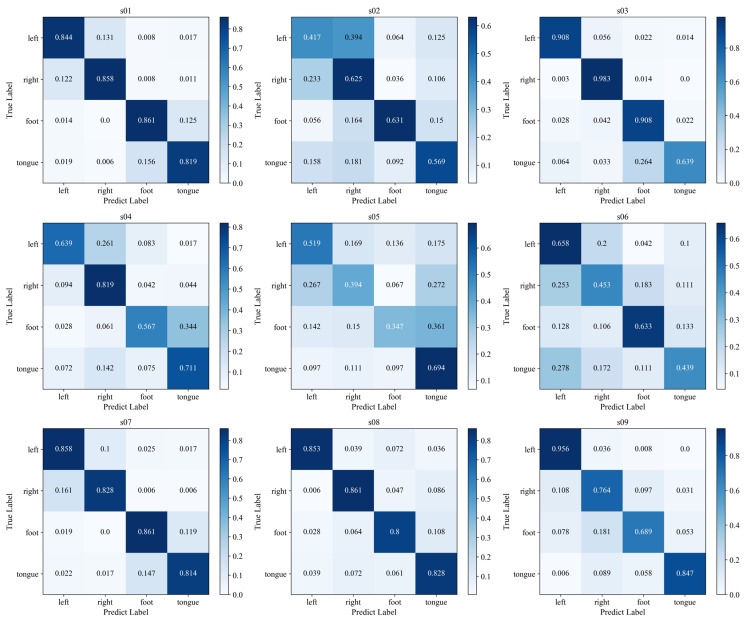
Subjects classification and recognition confusion matrix.

For the four classification data of motor imagery, in order to observe the classification performance of the proposed method on the four-class data of each subject, the confusion matrix of a motor imagery classification and recognition experiment of nine subjects is shown in Figure 4. It can be seen from Figure 4 that the classification recognition rates of the four categories of subjects s01, s07, and s08 are all above 80%. The tongue classification recognition rate of subjects s03 is relatively low, and the classification recognition rates of the left hand, right hand, and foot are all above 90. The foot classification performance of subjects s04 and s09 is relatively poor. The confusion matrix of subjects s02, s05, and s06 is relatively poor compared with other subjects, and it is easy to misidentify. However, for the whole dataset, the overall recognition results of the four categories are good. The results show that the method of adding sample transfer learning and small target data fine-tuning network in network model training has a better effect.

## 4. Conclusions

In this study, an attention mechanism-based multi-scale convolutional network is designed, and the transfer learning data alignment algorithm is introduced to analyze motor imagery EEG signals for improving the classification of motor imagery EEG signals. Experimental results showed that the average recognition rate of the proposed algorithm dual channel attention module migration alignment with convolution neural network (MS-AFM) reaches 80.76%, and the average classification result after the introduction of the transfer learning data alignment method is 86.03%, which is a significant improvement. In the experiment, five modes were compared: no sample transfer, direct sample transfer, sample transfer plus a small amount of target data fine-tuning, data alignment processing and sample transfer, data alignment processing and sample transfer, and a small amount of target data fine-tuning. The results show that the data alignment plus transfer learning Processing and fine-tuning with a small amount of target data have the highest classification accuracy, with an average recognition result of 86.0% for nine subjects, which is 5.3%, 9.4%, 3.4%, and 6.7% higher than the other four modalities, respectively. Furthermore, for the classification of motor imagery EEG signals, the direct sample transfer method without data alignment may reduce the classification accuracy, the sample transfer learning alignment method makes the EEG data distribution similar, and the sample isomorphic transfer learning can increase deep learning. The training set data to improve the classification accuracy of motor imagery EEG signals. At the same time, the EA method used in this paper is computationally simple and unsupervised, and can be used in more preprocessing situations.

## Figures and Tables

**Table 1:** Summary and comparison of transfer learning experiment results.

Evaluating Indicator (Evaluation)	Subject	Original	TL	TL + FT	EA + TL	EA + TL + FT
Acc ± Std (%)	S01	84.98 ± 1.17	77.85 ± 0.58	87.41 ± 1.78	82.39 ± 0.92	90.63 ± 1.13
	S02	69.86 ± 2.79	74.17 ± 0.79	70.38 ± 0.87	72.45 ± 0.58	79.23 ± 0.79
	S03	90.22 ± 1.11	81.64 ± 1.36	91.16 ± 0.98	87.48 ± 0.55	91.12 ± 0.88
	S04	78.97 ± 1.42	73.85 ± 0.92	80.34 ± 0.71	74.24 ± 1.17	84.16 ± 0.69
	S05	73.44 ± 1.62	74.35 ± 0.61	73.90 ± 0.71	73.69 ± 1.10	77.29 ± 1.00
	S06	74.86 ± 1.06	72.73 ± 2.85	78.55 ± 0.96	75.16 ± 0.77	79.03 ± 1.31
	S07	82.81 ± 0.97	75.14 ± 1.31	86.53 ± 1.20	77.30 ± 1.12	92.77 ± 0.86
	S08	84.91 ± 1.22	80.96 ± 1.66	87.35 ± 0.88	85.40 ± 2.31	90.87 ± 1.44
	S09	86.80 ± 1.32	78.94 ± 1.59	88.13 ± 0.97	85.77 ± 1.95	89.14 ± 1.09
	Avg	80.76 ± 0.89	76.62 ± 0.75	82.64 ± 0.46	79.32 ± 0.60	86.03 ± 0.55
Micro-F1 ± Std	S01	0.69 ± 0.026	0.61 ± 0.060	0.75 ± 0.034	0.65 ± 0.023	0.81 ± 0.025
	S02	0.34 ± 0.027	0.30 ± 0.035	0.35 ± 0.014	0.36 ± 0.012	0.56 ± 0.023
	S03	0.80 ± 0.027	0.70 ± 0.048	0.82 ± 0.022	0.75 ± 0.009	0.82 ± 0.018
	S04	0.55 ± 0.033	0.38 ± 0.041	0.58 ± 0.019	0.44 ± 0.033	0.67 ± 0.019
	S05	0.33 ± 0.023	0.27 ± 0.017	0.36 ± 0.025	0.41 ± 0.023	0.49 ± 0.028
	S06	0.43 ± 0.028	0.34 ± 0.049	0.51 ± 0.033	0.45 ± 0.019	0.54 ± 0.028
	S07	0.64 ± 0.029	0.43 ± 0.038	0.71 ± 0.031	0.52 ± 0.027	0.85 ± 0.017
	S08	0.69 ± 0.027	0.64 ± 0.057	0.73 ± 0.034	0.70 ± 0.054	0.81 ± 0.031
	S09	0.73 ± 0.031	0.53 ± 0.042	0.76 ± 0.021	0.71 ± 0.044	0.78 ± 0.024
	Avg	0.58 ± 0.013	0.47 ± 0.023	0.62 ± 0.011	0.55 ± 0.016	0.70 ± 0.012

## Data Availability

The authors sincerely thank BCI Competition IV Data sets 2a (https://www.bbci.de/competition/iv/#dataset2a, accessed on 25 April 2022) provided by the Institute for Knowledge Discovery (Laboratory of Brain-Computer Interfaces), Graz University of Technology, Graz, Austria (Clemens Brunner, Robert Leeb, Gernot Müller-Putz, Alois Schlögl, Gert Pfurtscheller) for their data providing.
